# Semi-Supervised Learning for Predicting Multiple Sclerosis

**DOI:** 10.3390/jpm15050167

**Published:** 2025-04-24

**Authors:** Sotiris Kotsiantis, Georgia Melagraki, Vassilios Verykios, Aikaterini Sakagianni, John Matsoukas

**Affiliations:** 1 Department of Mathematics, University of Patras, 26504 Patras, Greece; kotsiantis@upatras.gr; 2 Department of Military Sciences, Hellenic Army Academy, 16673 Athens, Greece; vgeorgiamelagraki@gmail.com; 3 School of Science and Technology, Hellenic Open University, 26335 Patras, Greece; 4 Intensive Care Unit, Sismanogleio General Hospital, 15126 Marousi, Greece; sakagianni@sismanoglio.gr; 5 Department of Chemistry, University of Patras, 26504 Patras, Greece; imats@upatras.gr; 6 Department of Physiology and Pharmacology, Cumming School of Medicine, University of Calgary, Calgary, AB T2N 1N3, Canada; 7 Immunology and Translational Research Group, Institution for Health and Sport, Victoria University, Werribee, VIC 3030, Australia; 8 NewDrug PC, Patras Science Park, 26504 Patras, Greece

**Keywords:** multiple sclerosis, semi-supervised learning, self-labeled techniques, medical prediction, machine learning

## Abstract

**Background**: Multiple Sclerosis (MS) is a chronic autoimmune disease of the central nervous system with a propensity to inflict severe neurological disability. Accurate and early prediction of MS progression is extremely crucial for its management and treatment. **Methods**: In this paper, we compare a number of self-labeled semi-supervised learning methods used to predict MS from labeled and unlabeled medical data. Specifically, we compare the performance of Self-Training, SETRED, Co-Training, Co-Training by Committee, Democratic Co-Learning, RASCO, RelRASCO, CoForest, and TriTraining in different labeled ratios. The data contain clinical, imaging, and demographic features, allowing for a detailed comparison of each method’s predictive ability. **Results and Conclusions**: The experimental results demonstrate that several self-labeling semi-supervised learning (SSL) algorithms perform competitively in the task of Multiple Sclerosis (MS) prediction, even when trained on as little as 30–40% of the labeled data. Notably, Co-Training by Committee, CoForest, and TriTraining consistently deliver high performance across all metrics (accuracy, F1-score, and MCC).

## 1. Introduction

Multiple Sclerosis (MS) is a complex, chronic autoimmune disease of the central nervous system that leads to cumulative neurological damage [1]. MS has a wide range of symptoms, from fatigue and motor dysfunction to cognitive dysfunction and visual disturbances, and thus early and accurate diagnosis is crucial for effective intervention. Traditional diagnostic procedures rely on clinical examination, magnetic resonance imaging (MRI), and cerebrospinal fluid examination, but these processes are time consuming and may be variable. Machine learning (ML) has been very effective in the field of medical diagnosis, with data-driven models that enhance prediction accuracy [2,3]. Of these, semi-supervised learning methods, which make use of labeled as well as unlabeled data, hold high potential for MS prediction, particularly in scenarios involving limited labeled medical data.

Self-labeled approaches, which involve semi-supervised learning, pseudo-label unlabeled data iteratively and enhance model performance progressively. Self-Training, Co-Training, and TriTraining are common approaches that employ varying mechanisms to refine predictions [4]. One classifier is utilized in Self-Training, which augments its training set with high-confidence predictions, while Co-Training applies several classifiers that transfer pseudo-labels through feature diversity. TriTraining extends the concept with three classifiers that cross-tag new instances, making them more capable of resisting noisy data. The viability of these methods in various applications in health has been shown before but not completely in terms of predicting MS. Cross-study comparisons between self-tagged methods could prove to be of great worth in terms of judging their suitability for MS-related data, where sampling tagged cases is still an issue within such data collection constraints.

This study provides a comprehensive comparison and evaluation of various self-labeled semi-supervised learning (SSL) approaches for the prediction of Multiple Sclerosis (MS) using a dataset comprising clinical, imaging, and demographic variables. By systematically assessing each method in terms of classification accuracy and generalizability across varying proportions of labeled data, we identify the most effective strategies for MS prediction under limited supervision. Our findings contribute to the growing body of research on SSL in medical contexts, highlighting the practical utility and efficiency of self-labeling techniques for supporting diagnostic decision-making, especially when annotated data are scarce or costly to obtain.

The findings of this study are also critical for personalized medicine, as accurate and timely prediction of Multiple Sclerosis (MS) disease progression can allow individualized treatment strategies to be developed for patients. By using semi-supervised learning models, healthcare providers can attain more precise risk stratification, allowing individualized therapeutic interventions based on a patient’s unique disease course. Furthermore, enhanced classification performance and the capacity to generalize such methods may be capable of assisting dynamic treatment adjustment based on data and, in turn, improve intervention effectiveness and ultimately patient outcomes in the treatment of MS.

The organization of this paper is as follows: Section 2 provides a critical overview of recent advances in the application of machine learning to predict Multiple Sclerosis. Section 3 provides an explanation of the dataset used in this study. The results derived from the application of the semi-supervised techniques described in Section 4 to the benchmark dataset are presented in Section 5. Finally, Section 6 and Section 7 provide a thorough discussion of the results and the conclusions based on this research.

## 2. Literature Review

The review [5] outlines the fundamentals of MS pathophysiology, pathogenesis, and diagnosis, and the categories of data used in computations, and summarizes the existing literature on the use of computer approaches such as MRI for a better understanding of MS.

The work [6] applies a machine learning platform to blood transcriptome and baseline brain MRI data to create prognostic models for Primary Progressive Multiple Sclerosis (PPMS). Predictions from RNA-sequencing data were based on PPMS patients in the ORATORIO trial and estimated disability progression and brain volume loss at 120 weeks. Models had 70.9% accuracy in predicting progression based on a 10-gene classifier and 70.2% in predicting brain volume loss based on a 12-gene model, which increased to 74.1% when using combined MRI information. A four-gene model distinguished further between rapid and slow progression. These findings substantiate the utility of blood transcriptome profiling for early risk stratification in PPMS.

The review [7] examined MRI-based ML for predicting MS progression, identifying the most significant biomarkers, and predicting disease conversion, cognitive impairment, and motor disability. Similarly, Moazami et al. [8] reviewed ML for applications in MS including automatic diagnosis, disease progression prediction, stage differentiation, and distinguishing MS from other diseases.

Wylezinski et al. [9] employed ML in the analysis of RNA-sequencing data to differentiate between neuromyelitis optica (NMO) and relapsing–remitting MS (RRMS) with a greater than 90% accuracy. Biomarkers for viral infection and ribosomal dysfunction inform potential drug targets, which include mitoxantrone and vorinostat.

To achieve subjective upper-limb measures in MS patients, Balaceanu et al. [10] normalized the Nine-Hole Peg Test (9HPT) with ML and neural networks, analyzing hand movement parameters to improve classification accuracy.

In [11], ML algorithms predicted MS disease activity, accrual of disability, and requirements for therapy based on clinical as well as biomarker data with increased accuracy when incorporating omics information. Similarly, Schultz et al. [12] discovered speech acoustics as objective measurements for MS as well as Friedreich ataxia, for which ML discriminative analysis segmented patient groups as a function of 21 indispensable acoustic features.

Tayyab et al. [13] compared probabilistic random forest ML models predicting MS conversion from clinically isolated syndrome to that of conventional models (AUC: 0.76, F1-score: 86.6%). Alternatively, Caba et al. [14] proposed an ML-based system to identify acute MS lesions in MRI, with a balanced accuracy of 74.3–74.6%.

Marzi et al. [15] used ML for the investigation of MRI markers that are linked with cognitive impairment in MS and found cortical gray matter and thalamus and hippocampus injury to be the major predictors. Similarly, Ciftci et al. [16] used ML on OCT data to identify demyelinating diseases in children with 75–80% accuracy.

Kenney et al. [17] set up ML-based MS and unilateral optic neuritis classification by OCT and visual function testing with 89% accuracy. Toosy et al. [18] presented a review of ML models for the discrimination of MS from controls and prediction of prognostic markers, with optic nerve involvement in prognosis.

Machine-learning-based spectral analysis in [19] can differentiate NMO from MS and controls with good sensitivity and specificity. MS disability progression was prognosticated via MRI markers in [20], with ML models based on T2 lesion load and thalamic volume being the most informative predictors.

Using MRI imaging information, Eshaghi et al. [21] identified three subtypes of MS—cortex driven, normal-appearing white matter driven, and lesion driven—directing risk stratification and response to treatment. Pontillo et al. [22] provided evidence for the utility of ML in predicting MS disability from routine brain MR images with high generalizability.

Kaur et al. [23] employed ML in gait analysis to diagnose MS, where regression normalization improved the classification accuracy. Similarly, Montolo et al. [24] employed clinical and OCT data to effectively predict MS diagnosis and disability worsening.

Predicting early MS cognitive status was examined in [25] using neuroimaging and ML, where the best-performing model was integrated over a single imaging modality (AUC: 0.90). Finally, Acquaviva et al. [26] used ML on transcriptomics data to diagnose MS, distinguish between disease stages, and offer demographic-independent accuracy.

## 3. Dataset

The information used in this study is from a prospective study conducted at the National Institute of Neurology and Neurosurgery (NINN) in Mexico City, Mexico, from 2006 to 2010. It comprises data collected from Mexican mestizo patients who had been newly diagnosed with clinically isolated syndrome (CIS), a disorder that often progresses to Multiple Sclerosis (MS). The primary objective of the original research was to identify predictors of CIS conversion to Clinically Definite Multiple Sclerosis (CDMS). The dataset, which was gathered by the curriculum of [27], provides valuable information on numerous clinical, demographic, and imaging-derived variables that have the potential to influence disease course. As early prediction is highly important in MS, analysis of these variables using machine learning techniques may make it easier to gain insight and enhance diagnostic accuracy.

The dataset contains a number of attributes that capture most important patient characteristics, consisting of pre-demographic variables such as gender, age, and years of schooling and clinical history variables such as past varicella infection and breastfeeding. The onset symptoms are categorized into visual, sensory, motor, and others in order to allow full description of the first presentations of patients. Other features are whether the CIS attack was polysymptomatic or monosymptomatic and whether oligoclonal bands are present in cerebrospinal fluid, a recognized MS biomarker. Several electrophysiological examinations, such as lower-limb somatosensory evoked potentials (LLSSEP), upper-limb somatosensory evoked potentials (ULSSEP), visual evoked potentials (VEP), and brainstem auditory evoked potentials (BAEP), provide further neurophysiological data. These points help to explain the heterogeneity of CIS presentations and their possible evolution to MS.

Furthermore, the dataset includes MRI findings, which are central to MS diagnosis and prognosis. Specific MRI features such as periventricular, cortical, infratentorial, and spinal cord lesions are recorded as binary indicators (positive or negative). Neurological disability is quantified with the Expanded Disability Status Scale (EDSS), and both the initial and final EDSS scores are taken into account to measure disease progression.

**Figure 1:**
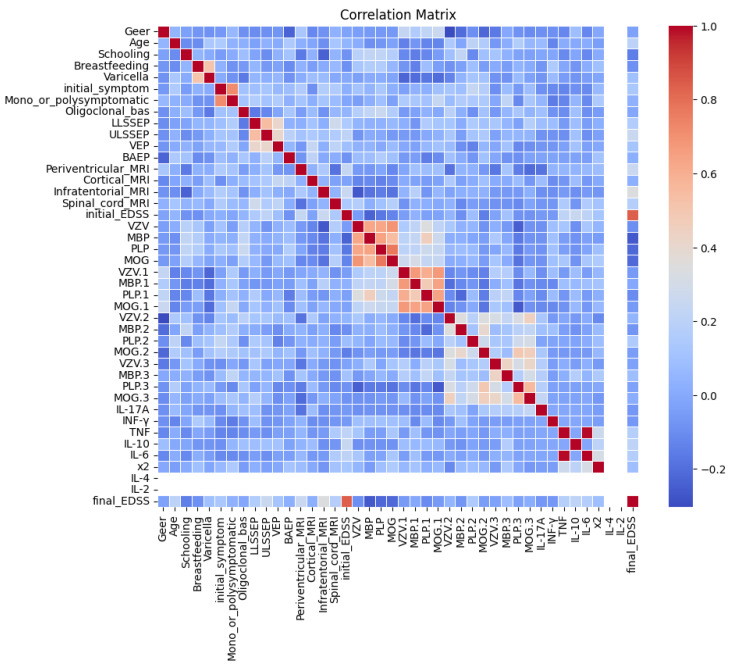
Feature correlation plot.

Use of a correlation plot (Figure 1) here is helpful in order to obtain insights about the relationships between features and detect potential dependencies or redundant information in the data. By displaying the correlation matrix as a heatmap, the analysis becomes more understandable when identifying highly correlated variables that can be problematic for multicollinearity in the model. It is very useful for showing magnitude and direction of correlations through color shades, and hence for detecting patterns visually.

**Figure 2:**
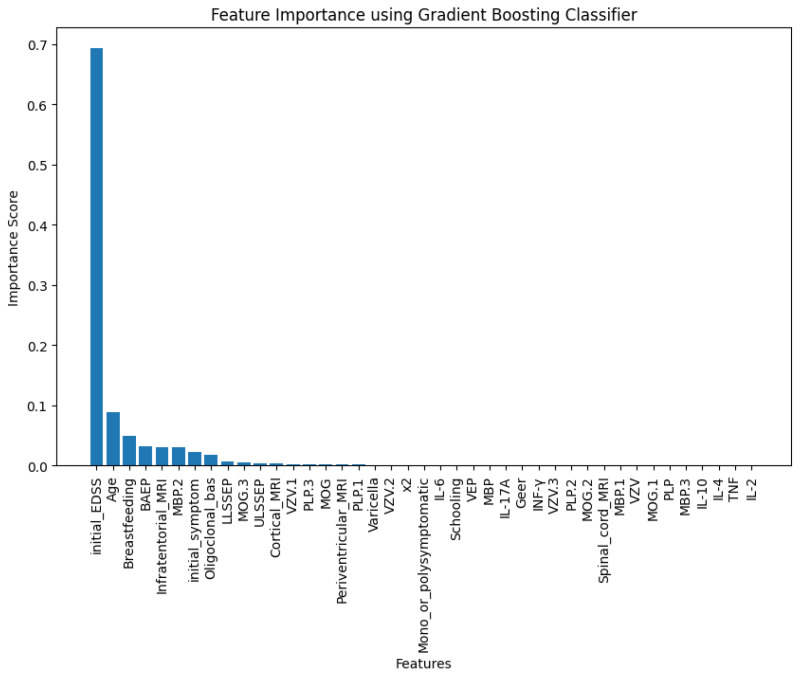
Feature importance score ranking.

The inclusion of a feature importance plot (Figure 2) in this study offers critical insights into the contribution of each variable to the prediction of Multiple Sclerosis. By leveraging the Gradient-Boosting Classifier [28], the analysis effectively identifies the most influential features, enabling a better understanding of the factors driving model predictions.

Our study aims to evaluate the usefulness of self-labeled semi-supervised learning approaches in predicting MS progression, which may be able to support early intervention and personalized treatment planning.

## 4. Semi-Supervised Algorithms

Self-labeling strategies are one class of semi-supervised machine learning methods that apply model prediction as pseudo-labels for unlabeled data. They are applied iteratively; the model is initially trained with a small labeled set, and its predictions over unlabeled samples serve as pseudo-labels for subsequent training. By mixing the original labeled data with the freshly generated pseudo-labels, the model is increasingly refined to possess improved performance. Self-labeling techniques prove most useful in the case of scarce or costly labeled data where the unlabeled data could be better exploited.

In this section, we present a brief overview of the self-labeling techniques used in our experiments.

Self-Training [4] is a semi-supervised learning approach in which a machine learning model continuously enhances itself based on its own prediction on unlabeled data. It initially trains the model on a limited labeled dataset. It then predicts the unlabeled data and selects the most confident among them as pseudo-labels. These pseudo-labeled samples are included with the original labeled dataset, and the model is re-trained to improve its generalization ability. The procedure can be repeated over multiple iterations to obtain even better performance. Judicious selection of the confidence threshold for pseudo-labels is nevertheless crucial because incorrect predictions can incorporate errors and negatively impact model accuracy.

SETRED (Self-Training with Editing) [29] and Self-Training are two semi-supervised learning methods that seek to use unlabeled data to enhance the performance of the model but differ in methodology and focus. Self-Training is a straightforward and usual technique whereby a model is initially trained on labeled data and later imposes pseudo-labels on unlabeled instances. These pseudo-labeled samples are incorporated into the training set in turn to update the model. Self-Training is, however, susceptible to failure in the event of noisy pseudo-labels. In contrast to Self-Training, SETRED adopts an ensemble-based solution to the latter problem through diversity of the models by virtue of regularized ensembling. SETRED avoids model overfitting to flawed pseudo-labels as a consequence of diversity in models, thus making the learning more robust. This makes SETRED particularly useful for complex tasks or datasets with noisy data or imbalance, whereas Self-Training is optimal for cleaner or less complex datasets due to its ease of implementation.

Co-Training [30] is a semi-supervised learning algorithm that employs multiple views or representations of the data to enhance learning performance. The idea is that various subsets of features, or “views”, have complementary information. Two models are initially trained separately using different sets of features on the labeled data. Each model then makes predictions on the unlabeled data, and the most confident predictions are transferred between models as pseudo-labels for subsequent training. The mutual transfer promotes learning by allowing each model to benefit from the superior parts of the other. Co-Training works well when the views are conditionally independent and contain sufficient information for predicting the target labels.

Co-Training by Committee [31] generalizes the Co-Training paradigm of semi-supervised learning to use an ensemble of multiple classifiers instead of two. In this approach, a committee of classifiers is initially established, each of which is trained on the labeled data from different subsets of features or from different learning algorithms. The classifiers then predict the unlabeled data, and the most confident predictions, that is, the ones concurred on by a subset of the committee, are used as pseudo-labels. Pseudo-labeled examples are part of the training set, allowing the committee to refine models gradually. By maintaining diversity among classifiers, the procedure enhances robustness, preventing the dangers of overfitting and error propagation. Co-Training by Committee excels in hard tasks when various views of the data provide complementary information.

Democratic Co-Learning [32] is a semi-supervised learning technique in which multiple classifiers work together to label unlabeled data through a democratic voting mechanism. Each classifier in the ensemble is trained on the same dataset but may differ in terms of learning algorithms, feature subsets, or initializations to ensure diversity. When assigning labels to unlabeled data, the classifiers vote on the most likely labels, and those supported by the majority are chosen as pseudo-labels for further training. This collaborative decision-making process helps mitigate individual model biases and reduces the risk of error propagation, a common issue in self-training approaches.

RASCO (Random Subspace Co-Training) [33] is a cutting-edge semi-supervised learning algorithm that generalizes the Co-Training strategy on the basis of robust pseudo-label reliability. Like traditional Co-Training, RASCO trains two classifiers on different feature subsets or views of the labeled training data, but it incorporates a measure of reliability for verifying the confidence of the pseudo-labels generated by each classifier before adding them to the other’s training set. This reliability mechanism prevents the propagation of erroneous pseudo-labels that contaminate model performance otherwise. By encouraging reliable pseudo-labels, RASCO enhances learning from sparse labeled data strength and efficiency, making it extremely well suited for processes such as text classification and other applications where data naturally might be separable into different views.

RelRASCO (Relevant Random Subspace Co-Training) [34] is a variant of the RASCO algorithm that further enhances the reliability of semi-supervised learning. Starting from the overall structure of RASCO, RelRASCO incorporates a reinforcement mechanism that automatically adjusts the pseudo-label reliability threshold in proportion to the learning progress of the classifiers. This adaptive strategy strikes a balance between exploration and exploitation such that high-quality pseudo-labels have the highest priority and the likelihood of incorporating noisy or incorrect labels is avoided. Additionally, RelRASCO accentuates iterated refinement, whereby reliability metrics improve as classifiers become refined, and hence it is particularly effective in learning from hard datasets with small numbers of labeled data.

CoForest [35] is a semi-supervised learning algorithm that uses the Co-Training concepts in combination with ensemble methods, namely decision forests. In the case of CoForest, multiple decision trees work together to classify unlabeled data based on the ensemble diversity. Each tree in the ensemble is individually trained on a portion of the labeled data. In the iteration process, the trees predict for the unlabeled set and select only the most confident predictions as pseudo-labels. The pseudo-labeled instances are appended to the training sets of the other trees to enhance mutual refinement. CoForest is especially effective in cases with scarce labeled data or sparse data, as the collaboration between trees enhances the robustness and generalization performance of the model.

TriTraining [36] is an ensemble semi-supervised learning technique that enhances classification precision by adding unlabeled information. It begins with training three classifiers on the original set of labeled instances. In each iteration, the classifiers predict labels for a subset of the unlabeled instances, and the pseudo-labeled instances are used to retrain the rest of the classifiers—provided the predictions concur. This agreement-based approach ensures that only reliable pseudo-labels are appended, thus preventing the possibility of error propagation. TriTraining is particularly beneficial when there are not many labeled data because it utilizes diversity among classifiers as well as data within unlabeled data to improve learning.

All the algorithms were run with the sslearn library [37], a Python library specifically for machine learning on semi-supervised data. The library enhances scikit-learn by offering additional tools and algorithms specific to semi-supervised learning tasks.

## 5. Results

**Figure 3:**
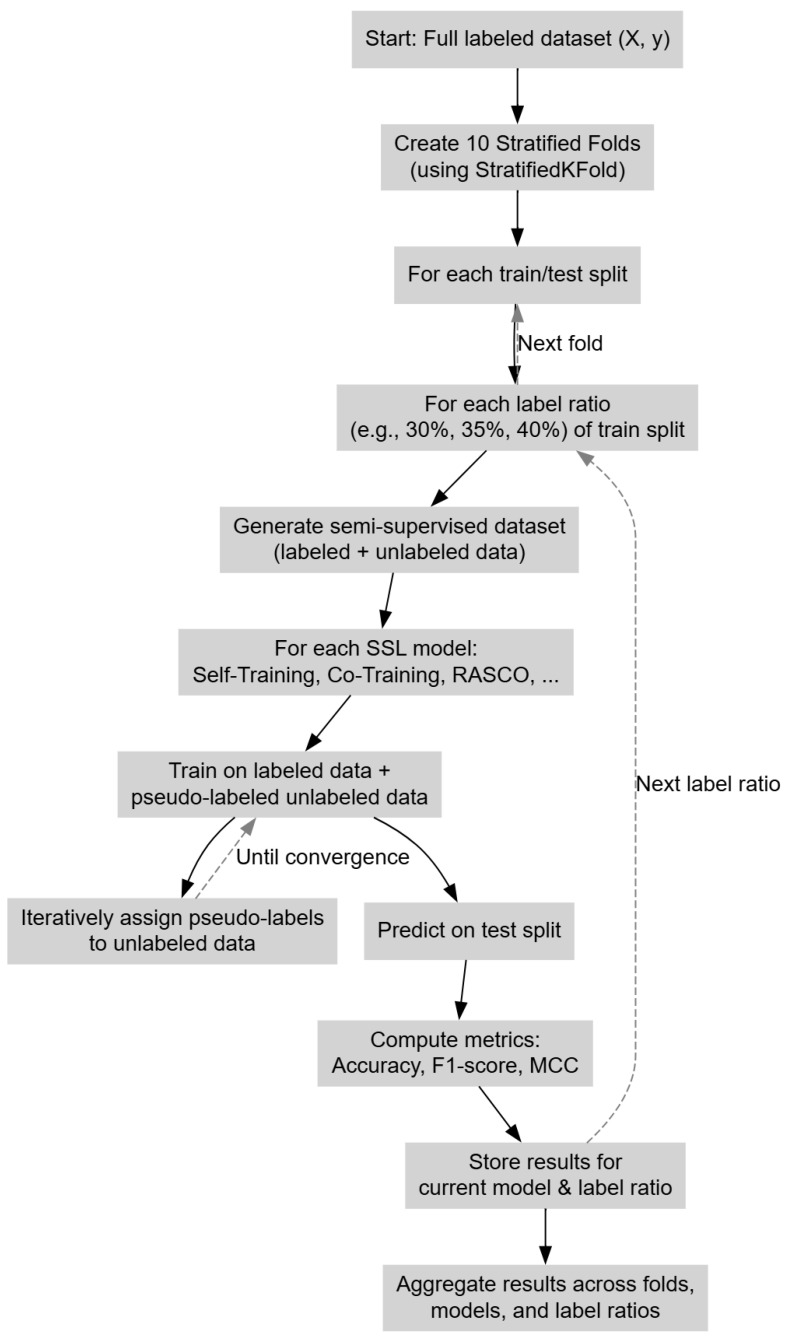
The experimental process.

To ensure a fair and reliable comparison of self-labeled techniques across different proportions of labeled data, we employed a structured cross-validation framework (Figure 3). For each label ratio setting (e.g., 30%, 35%, 40% of the data labeled), the dataset was first randomly partitioned into labeled and unlabeled subsets according to the specified percentage. The labeled subset was then subjected to ten-fold cross-validation: it was split into ten equally sized folds, with one fold serving as the test set and the remaining nine combined with the unlabeled subset to form the training set. This setup reflects a realistic semi-supervised scenario where only a portion of the data has known labels, and the model must leverage the larger pool of unlabeled data.

During training, self-labeled methods such as Self-Training, Co-Training, and RASCO iteratively assign pseudo-labels to the unlabeled data to improve performance. These pseudo-labeled instances are incorporated into the training process in each iteration. After training on each of the 10 folds, performance metrics such as accuracy, F1-score, and MCC are computed on the held-out test fold. The procedure is repeated for all 10 folds and for each label ratio, and the final results are reported as the average across all folds. This approach ensures a robust and unbiased evaluation of each method’s generalizability and performance under varying levels of label scarcity. All models were implemented using a Gradient-Boosting Classifier [28] as the base learner.

In this study, we largely employed the standard settings of semi-supervised classifiers in the SSLearn library as derived from earlier empirical testing. While thorough hyperparameter tuning was not the focus of this investigation, each algorithm was used with parameter values characteristic of its standard application and default behavior. For instance, SelfTrainingClassifier used a Gradient-Boosting Classifier as the base learner with pseudo-labels only being given when prediction confidence was more than 0.75 and a maximum of 10 self-training iterations. SETRED used a three-nearest neighbors classifier with a significance level of 0.05 for label rejection and limited training to 40 iterations, sampling 25% of the unlabeled data at each iteration.

Co-Training was initialized using a Gradient-Boosting Classifier and utilized 30 iterations of training for each run, selecting 75 unlabeled examples in every step, and implementing a confidence value of 0.5 to use when pseudo-labeling within an alternative feature–viewpoint configuration. Democratic Co-Learning used a decision tree classifier and favored label correction by applying a strong confidence constraint of 0.95 and utilized squared error weighting (with exponent = 2) in an attempt to balance label noise. RASCO used an ensemble of 30 Gradient-Boosting Classifiers, each using a random subspace with half the features, up to 10 iterations. CoForest used a seven-tree ensemble with bootstrapping and a decision threshold of 0.5. Finally, Co-Training by Committee used the default parameters of a BaggingClassifier ensemble, iterating to 100 times and sampling 100 from a pool of unlabeled samples per iteration to reach consensus-driven pseudo-labeling. These environments attempted to balance method fairness and complied with best practice as recorded in the literature.

**Figure 4:**
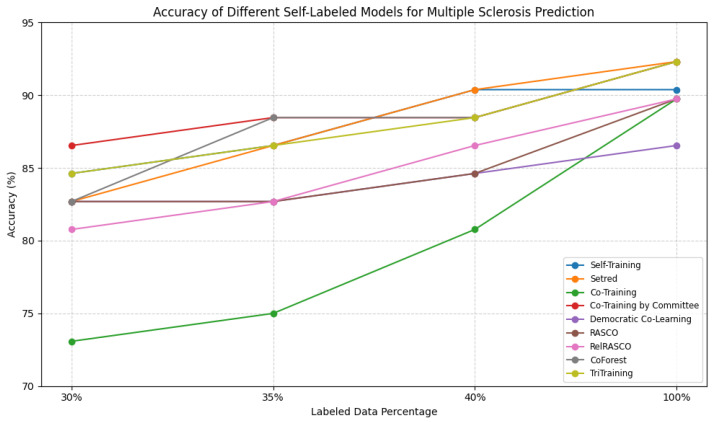
Accuracy of different self-labeled models across various percentages of labeled data.

The experimental result response presented in Table 1 and Figure 4 demonstrates the behavior of various self-labeled learning (SSL) algorithms in Multiple Sclerosis prediction with varied percentages of labeled data. Surprisingly, Co-Training yielded the poorest accuracy when trained on smaller portions of labeled data (30% and 35%), scoring only 73.08% and 75.00%, respectively. However, it performed substantially better when it was presented with a greater quantity of labeled data, reaching as high as 89.74% when fully supervised. In contrast, Co-Training by Committee and CoForest did very similarly well in all environments, with Co-Training by Committee doing the best of many others when there were few labeled samples.

Among the Self-Training-based algorithms, Self-Training itself remained at par throughout with 84.62% for 30% labeled data going up to 90.38% for 40% labeled data and maintaining performance even at 100%. SETRED performed similarly but had better results than Self-Training for fully labeled settings (92.31%). Democratic Co-Learning and RASCO had quite consistent performance but lagged behind the majority of the methods as far as absolute accuracy was concerned, with Democratic Co-Learning barely crossing 86.54% even with fully supervised data. At the same time, TriTraining and RelRASCO produced steady improvement with larger numbers of labeled data, with TriTraining producing the all-time highest accuracy (92.31%) for fully labeled setups.

In general, the results show that ensemble-based SSL algorithms such as Co-Training by Committee, CoForest, and TriTraining perform better than more primitive self-labeling algorithms, particularly when larger labeled sets are available. The relatively high-end accuracy in the majority of the models indicates that self-labeled learning is an excellent approach for Multiple Sclerosis prediction. Nevertheless, the performance difference between algorithms for lower percentages of labeled data underlines the importance of robust approaches that can take advantage of unlabeled instances in low-resource situations.

The F1-score and Matthews Correlation Coefficient (MCC) are both metrics used to evaluate classification models, but they emphasize different aspects of performance. The F1-score is the harmonic mean of precision and recall, focusing primarily on the positive class, and is especially useful when the cost of false negatives and false positives is high. On the other hand, the MCC provides a more balanced assessment by incorporating all four elements of the confusion matrix: true positives, true negatives, false positives, and false negatives.

From the perspective of robustness and balanced performance, RelRASCO and CoForest stand out in terms of F1-score (Table 2) and the MCC (Table 3), with RelRASCO achieving the highest MCC of 76.03% under full supervision, indicating a strong correlation between predicted and actual classes even in imbalanced settings. Meanwhile, Democratic Co-Learning and RASCO showed moderate performance, with RASCO suffering in terms of the MCC despite relatively acceptable accuracy values. Co-Training, though conceptually simple, consistently underperformed across all metrics, highlighting the limitations of using two classifiers trained on disjoint feature views in this context. Overall, the results validate the effectiveness of SSL techniques, particularly ensemble-based methods, in leveraging limited labeled data for reliable MS prediction.

**Table 2:** F1-score of different self-labeled models for Multiple Sclerosis prediction (Mean ± Std).

SSL Algorithms	30%	35%	40%	100%
**Self-Training**	74.24 ± 1.86%	75.14 ± 1.88%	77.17 ± 1.93%	79.59 ± 1.99%
**SETRED**	74.83 ± 1.87%	76.64 ± 1.92%	77.26 ± 1.93%	79.29 ± 1.98%
**Co-Training**	67.44 ± 1.69%	70.46 ± 1.76%	70.46 ± 1.76%	76.39 ± 1.91%
**Co-Training by Committee**	79.59 ± 1.99%	80.66 ± 2.02%	81.37 ± 2.03%	82.37 ± 2.06%
**Democratic Co-Learning**	71.49 ± 1.79%	78.28 ± 1.96%	76.37 ± 1.91%	76.37 ± 1.91%
**RASCO**	68.13 ± 1.70%	65.06 ± 1.63%	73.73 ± 1.84%	73.73 ± 1.84%
**RelRASCO**	75.57 ± 1.89%	83.87 ± 2.10%	83.87 ± 2.10%	83.87 ± 2.10%
**CoForest**	76.82 ± 1.92%	81.11 ± 2.03%	83.10 ± 2.08%	83.10 ± 2.08%
**TriTraining**	76.83 ± 1.92%	76.83 ± 1.92%	80.95 ± 2.02%	81.86 ± 2.05%

**Table 3:** MCC score of different self-labeled models for Multiple Sclerosis prediction (Mean ± Std).

SSL Algorithms	30%	35%	40%	100%
**Self-Training**	57.69 ± 1.44%	57.69 ± 1.44%	62.26 ± 1.56%	65.79 ± 1.64%
**SETRED**	57.95 ± 1.45%	61.90 ± 1.55%	61.90 ± 1.55%	65.52 ± 1.64%
**Co-Training**	40.38 ± 1.01%	45.54 ± 1.14%	51.75 ± 1.29%	60.41 ± 1.51%
**Co-Training by Committee**	69.00 ± 1.73%	69.00 ± 1.73%	69.00 ± 1.73%	69.03 ± 1.73%
**Democratic Co-Learning**	62.13 ± 1.55%	64.60 ± 1.61%	64.60 ± 1.61%	64.60 ± 1.61%
**RASCO**	44.22 ± 1.11%	46.32 ± 1.16%	57.79 ± 1.44%	62.83 ± 1.57%
**RelRASCO**	63.74 ± 1.59%	67.13 ± 1.68%	68.63 ± 1.72%	76.03 ± 1.90%
**CoForest**	63.24 ± 1.58%	65.58 ± 1.64%	71.18 ± 1.78%	71.18 ± 1.78%
**TriTraining**	53.81 ± 1.35%	58.66 ± 1.47%	66.93 ± 1.67%	66.93 ± 1.67%

## 6. Discussion

This study on applying semi-supervised learning methods for predicting Multiple Sclerosis (MS) was triggered by earlier extensive research of ours on the development of therapeutic vaccines against MS with an ultimate target of personalized treatment [38]. Our previous studies have been focused in the design, synthesis, and in vitro and in vivo study of myelin epitope peptides (MBP 83-98, MOG 35-55, PLP 39-55) in linear, cyclic, and mannan-conjugated forms. From several potent candidate compounds, Mog35-55 conjugated with mannan through Linker (KG)5 was selected for clinical trials, which are currently at the phase 1 stage [38,39,40]. Earlier cutting-edge synthetic studies [41,42] were the basis of the current clinical trials.

The results of this study align with findings from previous work on self-labeled learning (SSL) methods for medical diagnosis, complementing the effectiveness of ensemble-based methods. Similarly to previous experiments, where Self-Training and SETRED were established to be very successful in semi-supervised learning, our findings confirm their strong performance, particularly when there are more labeled data. However, the performance gains observed with Co-Training by Committee, CoForest, and TriTraining suggest that the ensemble approaches would give a more generalized performance in complex medical classification tasks. In contrast to methods like Co-Training and Democratic Co-Learning, which were relatively decent in earlier works, these ensemble methods are proven to deteriorate with fewer examples to label, showing flaws within their self-labeling strategies for this specific database.

One of the major differences from earlier work is the inconsistency in the gains in performance at different levels of labeled data. Whereas earlier experiments hinted at a linear rise in accuracy as the labeled set grows, our findings reveal that certain algorithms like Self-Training and SETRED plateau faster, whereas others like CoForest and TriTraining keep improving with more labeled data. This means that the choice of SSL algorithm has to be dependent on data, as various models respond differently to varying levels of supervision.

## 7. Conclusions and Future Work

In this work, the ability of a variety of semi-supervised learning (SSL) algorithms to classify Multiple Sclerosis was investigated and it was found that data-based self-labeled ones are capable of considerably enhancing classification accuracy even when minimal labeled data are employed. It also confirms the performance benefit of ensemble-based SSL algorithms, such as Co-Training by Committee, CoForest, and TriTraining, which were consistently better than individual self-labeling strategies. Although Self-Training and SETRED performed optimally on different label ratios, their improvement in accuracy was capped ahead of more advanced ensemble methods. These findings highlight the promise of SSL for medical diagnosis where access to highly labeled data is generally costly and time consuming.

In addition to its ability to enhance classification accuracy, SSL also holds promise for advancing personalized medicine. Utilizing both labeled and unlabeled samples, SSL algorithms can facilitate more personalized MS progression prediction, enabling clinicians to better tailor treatment protocols to each patient’s unique disease profile. Having the ability to attain maximum predictive performance with fewer labeled samples, SSL can facilitate the development of personalized risk profiles, which can lead to earlier intervention and more accurate therapeutic decisions.

Overall, the study confirms that SSL can be an effective alternative to fully supervised learning for Multiple Sclerosis prediction, reducing reliance on large labeled datasets without sacrificing predictive accuracy. However, variations in SSL model performance mean that algorithm choice must be driven by data availability and structure. There should be follow-up studies to optimize SSL methods for medicine that involve combining domain knowledge, continuing optimization of self-labeling algorithms, and examining hybrid methods that possess the strengths of multiple SSL approaches. This additional optimization might elevate prediction to an even higher level, finally ending in the incorporation of SSL within personalized medicine frameworks and improving patient care with greater accuracy and tailor-made clinical decision-making.

For future research, we plan to expand this study by using more advanced feature selection methods and investigating deep semi-supervised learning models. Self-supervised pretraining or contrastive learning of neural networks may further improve the performance of SSL methods, especially for complicated medical data. Moreover, incorporating domain knowledge by expert-driven feature engineering or reinforcement learning may enhance model interpretability and clinical applicability. Finally, the verification of these models in larger and more representative patient groups will be essential for ascertaining their generalizability and utility to actual clinical practice in the diagnosis and prognosis of MS. Through the development of SSL methods and the rectification of their existing shortcomings, we aim to render machine learning prediction for neurological disease more accessible and reliable.

## Figures and Tables

**Table 1:** Accuracy of different self-labeled models for Multiple Sclerosis prediction (Mean ± Std).

SSL Algorithms	30%	35%	40%	100%
**Self-Training**	84.62 ± 2.12%	86.54 ± 2.16%	90.38 ± 2.26%	90.38 ± 2.26%
**SETRED**	82.69 ± 2.07%	86.54 ± 2.16%	90.38 ± 2.26%	92.31 ± 2.31%
**Co-Training**	73.08 ± 1.83%	75.00 ± 1.88%	80.77 ± 2.02%	89.74 ± 2.24%
**Co-Training by Committee**	86.54 ± 2.16%	88.46 ± 2.21%	88.46 ± 2.21%	92.31 ± 2.31%
**Democratic Co-Learning**	82.69 ± 2.07%	82.69 ± 2.07%	84.62 ± 2.12%	86.54 ± 2.16%
**RASCO**	82.69 ± 2.07%	82.69 ± 2.07%	84.62 ± 2.12%	89.74 ± 2.24%
**RelRASCO**	80.77 ± 2.02%	82.69 ± 2.07%	86.54 ± 2.16%	89.74 ± 2.24%
**CoForest**	82.69 ± 2.07%	88.46 ± 2.21%	88.46 ± 2.21%	92.31 ± 2.31%
**TriTraining**	84.62 ± 2.12%	86.54 ± 2.16%	88.46 ± 2.21%	92.31 ± 2.31%

## Data Availability

The dataset can be found in [27].
